# Integration of Smart Piezoelectric Devices into Robotic and Wearable Medical Systems Driven by Smart Digital Tools

**DOI:** 10.3390/mi17101176

**Published:** 2026-10-10

**Authors:** Adel Razek

**Affiliations:** Group of Electrical Engineering–Paris (GeePs), CNRS, University of Paris-Saclay and Sorbonne University, F91190 Gif sur Yvette, France; adel.razek@centralesupelec.fr

**Keywords:** biomedical tools, piezoelectric actuation and sensing, robotic interventions, wearable devices, health status tracking, active assistive tools, health monitoring

## Abstract

This narrative review aims to analyze and highlight the potential of piezoelectric biomedical devices for actuation and sensing tasks, enabling reliable, high-precision results in innovative applications driven by smart digital tools. This encompasses high-precision actuation for robotic interventions, as well as observation, monitoring, and organic function support tasks using wearable sensors and actuators. The nature of these applications drives the development of high-precision technological tools, highly accurate medical robotic operations, and reliable wearable devices. Precision operations involve robotic structures actuated directly by integrated piezoelectric materials—configured as traveling-wave beams or plates—or robotic joints actuated by piezoelectric deformation converted into motion. These devices can perform surgical interventions or facilitate targeted drug delivery. As for wearable devices, they are placed in close proximity to the body or integrated directly into it. They can perform passive or targeted tasks through interaction with body tissues. They provide sensing, recognition, monitoring, and control functions—thereby contributing to diagnosis, health status tracking, and prognosis—as well as active assistance functions, such as stimulating organic functions or aiding limbs with sensorimotor impairments. The article examines medical devices intended for sensing, monitoring, assistance, and intervention; sensors and actuators based on smart materials; piezoelectric-based robotic and wearable medical systems; and their integration into autonomous medical systems driven by smart digital tools. The various themes of this narrative review, while distinct, are supported by examples from the literature.

## 1. Introduction

Humanity has forever pursued good in conditions of security, health and well-being. The position of healthcare in this perspective has progressed solidly, including surveillance and disclosure, alongside medication via assistive therapeutic or medical interventions, see for example, cases of ancient and traditional medicine [1,2,3]. Such actions are linked to committed apparatuses and specialized competences of health check staff. Certainly, surveillance and disclosure efforts regarding various sizes, for instance heart rate, body temperature and more, permit staff to identify and see coming health troubles and conclude which body part requires assistance—such as stimulation, backing, etc.—or interventional act, such as the administration of medication or a surgical procedure.

Originally, monitoring relied primarily on human sensory faculties—such as the perception of sound, sight, or touch—to detect internal abnormalities with the aid of external tools (like the stethoscope for hearing or a light source for vision). Similarly, interventions involved invasive incisions performed using manually operated instruments and external artificial lighting. Although these practices remain useful, they can now be advantageously replaced in many situations.

Throughout history, the healthcare sector has advanced toward an ultimate goal: ensuring patient safety and optimizing the performance of medical personnel. Consequently, modern medical devices designed for monitoring, detection, and assistance must be minimally invasive—or non-invasive—while delivering high precision and real-time operation. Interventional surgical devices and those used for drug delivery are subject to these same requirements, with the additional need for increased precision in positioning and movement, thereby enabling treatment to be confined to the targeted tissue area while sparing healthy tissue [4].

Recent technological progresses in sensors, actuators, and digital or image-guided robotics have fostered overall advancement in the fields of medical monitoring, detection, assistance, and interventions. The use of body-centric wearable sensors [5] and assistive devices [6,7,8], as well as the robotization of interventions and self-sufficient digital or image-guided processes [9], are directly linked to these technological developments.

Furthermore, the aforementioned technical advances are associated to the combined use of smart materials [10,11] and intelligent digital management systems [9], thereby contributing significantly to patient well-being and easing the workload of staff. This smart environment is revolutionizing the medical system [12,13]. It encompasses the use of smart materials in wearable sensing and assistive devices as well as in robotic actuation, alongside intelligent digital management tools for interventional medical procedures. Moreover, this intelligent digital management empowers the personalization of interventions through precision medicine [14]; this has transformed processes for identification, handling, and medication instruction while offering enhanced precision. Finally, beyond therapeutic efficacy, this approach helps reduce common place side effects and fosters the deployment of digital health technologies [15]. Besides, from a sustainability perspective, the relevant components within this smart background must be defended against electromagnetic (EM) radiation, which is virtually omnipresent in today’s everyday applications [16]. This protection can be achieved through the use of ingredients immune to EM interference (EMI) or smart shielding systems [17].

Regarding the use of smart materials in wearable medical devices and interventional robotic actuation, piezoelectric-based sensors and actuators are prime candidates, given the specific requirements of these applications—such as wearable sensing tools [18] or systems for interventional robotic surgery and drug delivery [4]. In general, positioning and displacement operations are common in state-of-the-art precision equipment, robotic mechanisms, and similar systems. These tasks can be performed by actuators offering high positioning accuracy and fine motion resolution, alongside other specific characteristics. Furthermore, reliable system health monitoring is enabled through the precise sensing of different parameters. In this background, piezoelectric actuators and sensors stand out as ideal solutions, particularly for robotic applications [19,20,21], sensing [22], and, more broadly, sensing or actuation functions [23,24,25].

The aforementioned intelligent digital management systems rely on autonomous processes governed by feedback loops and operate under staff supervision, thereby fostering personalized medical care and efficient human-machine interaction. The consistency of process control depends on process complexity, uncertainties associated with the parameters involved, and unanticipated external bullying. These factors can be managed through process monitoring using intelligent digital tools such as the digital twin (DT) [26,27]. Furthermore, the use of digitally and intelligently augmented processes—to facilitate medical monitoring, detection, assistance, and interventions—has become a priority focus. This entails the use of artificial intelligence (AI) [28,29,30] and extended reality (xR) [31,32,33]. The use of these intelligent digital tools allows, among other things, a reduction in data processing volume, process optimization, targeted medical therapies, more precise intervention planning, training, and treatment adaptation, all while reshaping care models—whether conventional or personalized. The implementation of these intelligent digital tools results in intelligent digital monitoring.

The aim of this narrative review is to analyze and highlight the potential of piezoelectric biomedical devices for actuation and sensing functions, enabling reliable, high-precision results in innovative medical applications driven by smart digital tools. These applications range from high-precision actuation for medical robotics interventions to the monitoring and control of various physical phenomena using wearable sensors and actuators designed for sensing and assistance. Such reliable and precise tools are closely linked to patient safety and ease of use for medical personnel. The paper specifically examines:

- medical devices for detection, monitoring, assistance, and intervention;
- actuators and sensors based on smart materials in medical applications;
- piezoelectric robotic and wearable medical systems;
- integration into medical systems driven by intelligent digital tools.

Numerous studies have been published on piezoelectric biomedical devices designed for actuation and sensing functions. This article builds upon and complements this prior work by providing a key synthesis of analyses regarding the potential of these devices to deliver reliable, high-precision results in innovative medical applications, supported by intelligent digital tools. The topics covered are based on carefully selected references derived from either targeted research or alerts configured for specific emerging themes. Critical comparisons of the various technologies involved are presented in the cited review articles. The selection of all references was guided by content requirements, thereby avoiding textual repetition within the manuscript. Figures reproduced from external sources are governed by a Creative Commons CC BY open license, with the source duly acknowledged.

**Figure 1:**
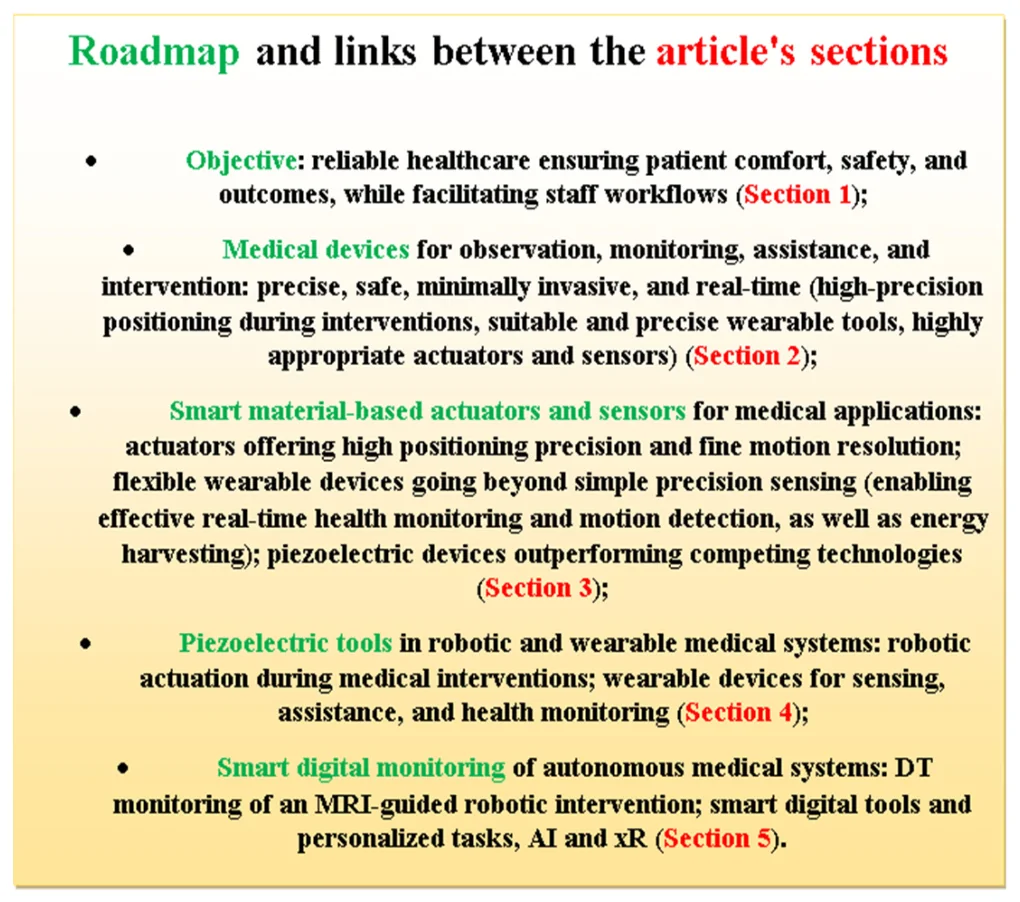
Roadmap of the article and the links between the various sections.

Figure 1 presents a general roadmap of the article, as well as the links between the various sections.

## 2. Medical Devices for Monitoring, Assistance, and Intervention

As previously noted, the healthcare sector has seen steady progress—particularly in screening and monitoring, as well as through the use of supportive therapies or medical interventions. These in progress healthcare areas rely on specialized devices designed to detect and monitor various conditions; they enable the identification of potential future issues and the determination of necessary assistance or intervention for the affected organs, while utilizing the specific devices dedicated to these procedures. Two categories of devices play a role in this modern medicine: wearable therapeutic devices and interventional devices. The first category encompasses detection, monitoring, and assistance, while the second focuses primarily on robotic procedures such as, surgery, embedded drug delivery, and assistive tasks. These two categories can operate in combination; for example, a wearable assistance device may rely on a robotic assistive procedure. Furthermore, although both categories may utilize sensors and actuators, these components are not employed for the same tasks in each category.

### 2.1. Wearable Therapeutic Devices

Wearable devices are tools—whether handheld, detachable, or implanted—that are in direct contact with the body or integrated into it. They can perform passive or directed tasks involving tissues, such as sensing or assistive actions; these operations can be distinct or coordinated, autonomous or remotely controlled, and carried out continuously and in real time. Furthermore, the tasks performed by wearable devices—such as monitoring, tracking, prediction, and stimulation—enable the organization of post-treatment care following the initial disturbances. Thus, by eliminating the constraints associated with patient repositioning, travel, or transfer, a combined and connected care methodology replaces in-person rehabilitation.

Detection wearable duties can utilize pressure sensors, for instance different applications [34,35,36,37,38] or incorporated antennas biosensors, for example [39,40,41,42,43], which offer advantage of consent to patients to achieve their daily realizations while staying supervised. Assistive wearable functions include tissue-incorporated devices for health restores and diverse organs stimulation, and assisting devices for body limbs with sensorimotor insufficiencies. These assistive functions may include, for example, stimulation by pacemaker and defibrillator [44], flexible drug delivery via implant [45] monitoring via spinal cord implant [46], cardiac blood flow assistance [47], robotic lumbar spinal injection [48], and assistance of limbs with sensorimotor deficiencies [6,7,8].

It is now possible to utilize wearable devices offering optimal performance fitting their projected execution, thanks to current digital intelligence technologies [13] and smart materials [24].

It is worth noting that a material suitable for wearable devices would be capable of performing multiple functions and enabling the design of flexible equipment. These characteristics of multifunctionality and flexibility are linked to human body movements; thus, a suitable material would adapt to such movements through its flexibility and could harness the resulting kinetic energy via an energy-harvesting function that complements its sensing and actuation capabilities.

### 2.2. Medical Interventional Procedures

Medical interventions primarily involve robotic surgery and assisted drug delivery procedures [4,9]. It should be noted that the latter differ from the previously mentioned flexible, implantable drug delivery systems, which are associated with wearable devices and do not require medical intervention [45]. Surgery and assisted drug delivery procedures alike aim to combine safety and precision, thereby ensuring the patient comfort essential to well-being. Success depends on factors such as the precision of the mechanism and positioning, the degree of tissue invasiveness, the duration of the procedure, and the speed of recovery, as well as on the vigilance of medical staff, ergonomics, and ease of execution. In addition, a crucial challenge lies in precisely limiting the intervention to the worried zone, in this way safeguarding healthy bordering tissues [4,9].

The potential of autonomous, secure, and minimally invasive intervention—using devices that substitute for the visual and perceptual capabilities of personnel while remaining under their full supervision—is now achievable owing to topical digital intelligence technologies [13], particularly robot-assisted procedures (involving computing or imaging) [9], that utilize tools actuated by smart materials [24].

## 3. Actuators and Sensors Based on Smart Materials in Medical Uses

As previously mentioned, positioning and displacement are common operations in advanced robotic mechanisms. These tasks can be performed by actuators offering high positioning precision and fine motion resolution, while also providing other advantages: rapid response times, high stiffness and actuation force, simple structure, flexible travel range, resistance to EMI, and compactness. Furthermore, the precise detection of various parameters plays a crucial role in monitoring a wide range of mechanical and physical phenomena. In this context, as previously indicated, piezoelectric actuators and sensors show up as ideal solutions, particularly for robotic applications [19,20,21], sensing [22], and general sensing or actuation functions [23,24,25].

Regarding actuators, various technologies are available; the most conventional types are hydraulic, pneumatic, or EM, alongside those utilizing smart materials such as piezoelectric materials, electroactive polymers, magnetostrictive materials, shape-memory alloys, and photomechanical materials. Their functional distinctiveness lies in the conversion of energy into motion, and they offer specific characteristics such as power output, response time, precision, stiffness, stroke flexibility, and suitability for the operating environment. For the medical applications in question, conventional hydraulic and pneumatic actuators suffer from limiting response times, while EM actuators are vulnerable to EMI. Given these shortcomings and the rapid rise of advanced medical technologies requiring high-precision positioning, piezoelectric actuators stand out due to their nanometric resolution, rapid response, and immunity to EMI, thereby outperforming other smart technologies such as shape-memory alloys and magnetostrictive actuators [49,50,51]. Furthermore, their structural scalability and exceptional load-bearing capacity make them preferred solutions in the healthcare sector.

Table 1 presents a comparison between piezoelectric actuators and traditional or smart actuation methods (electromagnetic, hydraulic, or shape-memory alloy) in terms of resolution, response time, actuation force, and MRI compatibility [24].

In the field of wearable devices, beyond precision sensing, flexible and malleable piezoelectric materials—such as polyvinylidene fluoride (PVDF) and flexible piezoelectric composites (FPC)—enable effective real-time health monitoring, motion detection, and energy harvesting from biomechanical movements, all of which can be integrated into smart textiles [52,53,54].

## 4. Piezoelectric Tools in Robotic and Wearable Medical Systems

As previously noted, recent medical advances have made it possible to manage various conditions—starting at the diagnostic stage—through therapeutic support and medical interventions. The effectiveness of this care is closely linked to strategies focused on patient safety and comfort, as well as on achieving optimal outcomes, prioritizing minimally invasive (MI) and rigorously monitored procedures. In this context, two main types of care require piezoelectric devices: robotic actuation during medical interventions and sensing via wearable devices for health monitoring. Furthermore, other treatments directly utilize piezoelectric materials, such as piezoelectric intervention [55] and hyperthermia using ultrasonic transducers [56].

### 4.1. Robotic Actuation in Medical Interventions

For targeted medical applications, piezoelectric robotic actuation can be achieved using self-propelled miniature structures that incorporate the piezoelectric material, or via joint actuators that convert piezoelectric deformation into indirect motion.

#### 4.1.1. Self-Propelled Miniature Robotic Structures

**Figure 2:**
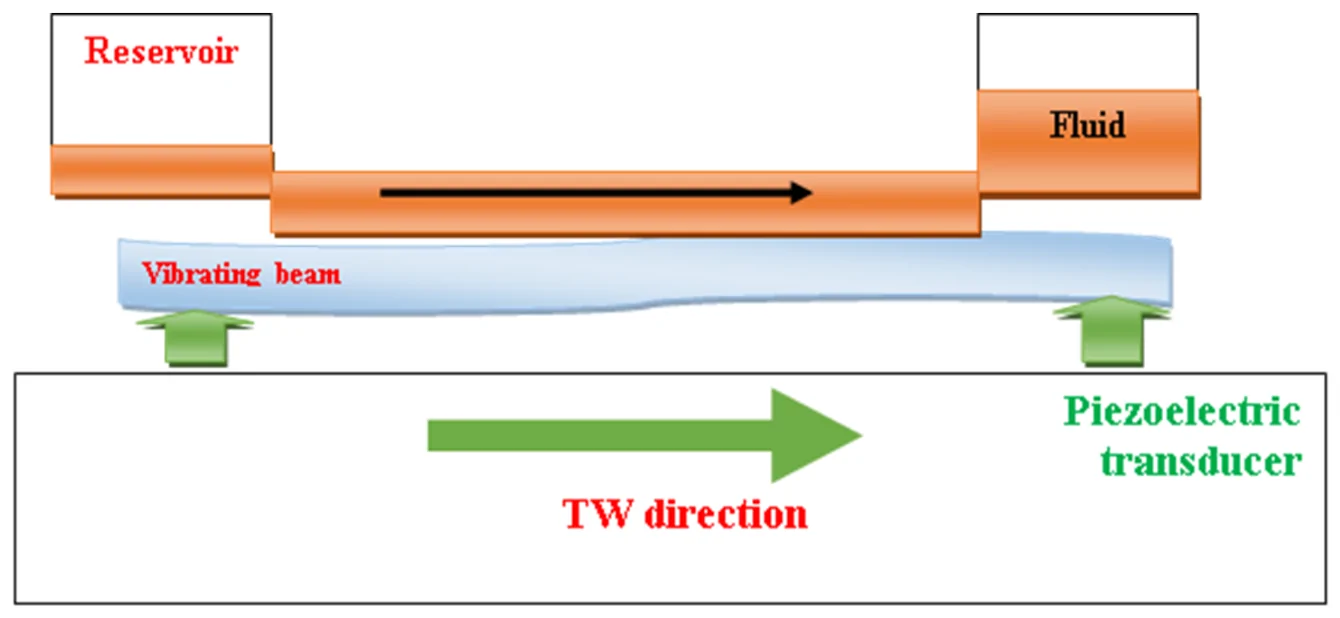
Schematic of the operating principle of a TW mini piezoelectric pump that moves a fluid at a precisely controlled flow rate [12].

The category of self-propelled robotic structures generally encompasses traveling-wave (TW) beams or plates used in applications involving the controlled, precise movement of relatively light loads across a surface [21,57,58,59] or the transport of fluids within conduits, as is the case with micropumps [20,60,61]. These autonomous robotic structures are employed in precision medical tasks requiring reliability, repeatability, a wide range of motion, and smooth movement. For example, Figure 2 shows a schematic of the operating principle of a TW piezoelectric mini pump that moves a fluid at a precisely controlled flow rate [12,20,61].

Against the backdrop described above, various manufacturing and assembly approaches have been developed to create these autonomous piezoelectric robotic structures in diverse forms (beams, plates, rods, conduits, etc.) and incorporating different types of piezoelectric actuation (bonded, loaded, integrated, etc.). The bonded-element structure—favored for its compatibility with flexible, widely accessible mechanical designs—has driven strong demand for precision medical robotics tools. Promising prospects are thus emerging in the field of miniature robotics, particularly regarding increased degrees of freedom, enhanced transmission and positioning capabilities [62], an extended range of speeds and torques [63], superior operational travel and driving force [64], millisecond-range response times, millinewton-range output forces, micrometer-scale resolution [65], and centimeter-range reconfigurable robots [66].

Furthermore, miniature piezoelectric robotic structures are particularly well-suited for navigating complex environments—such as the interior of the human body—thanks to their lightness and flexibility; they can thus enable drug delivery and disease identification. Moreover, their carrying out is frequently established on bio-inspired concepts. For example, a robotic structure consisting of a thin, flexible plate actuated by precisely positioned piezoelectric patches [21,59] can replicate surface crawling, flying, or swimming movements. The literature presents various instances of bio-inspired piezoelectric actuation, such as a multi-legged robot [67], as well as other examples drawing inspiration from the earthworm, rhinoceros beetle, locust, rotary gallop gait, squirrel gallop, octopus crawling, and swinging movement of jellyfish, described in references [68,69,70,71,72,73,74], respectively.

#### 4.1.2. Actuated Robotic Joints for Medical Interventions

This section is dedicated to the second category of robotic actuation, which employs joint actuators that convert piezoelectric deformation into indirect motion. This type of actuation applies to all medical robotic interventions, although the strategy adopted varies depending on the nature of the procedure; therefore, the characteristics of the various interventional medical procedures will be presented first.

In recent times, medical intervention methods have progressed substantially and continue to evolve through open surgery, laparoscopy, and various types of robotic procedures. This evolution has brought various profits to patients, yet the complexity of interventions has increased, and the role of personnel has changed considerably [75].

Open surgery is a common, enduring practice; however, its invasive nature entails various risks, and MI procedures are preferred whenever possible. Laparoscopic procedure offers patients different benefits thanks to its MI technology [76,77], providing an expanded view via a camera coupled with a light source [78,79]. Furthermore, it is associated with reduced postoperative discomfort—and consequently a shorter duration of clinical care—as well as better cosmetic outcomes and a lower risk of long-term sequelae [78,79,80], while also enabling diagnostic laparoscopy [81]. Conversely, the use of long instruments combined with two-dimensional visualization can lead to ergonomic issues during the procedure [76,82] as well as potential postoperative risks [83]. Robot-assisted laparoscopy helps mitigate some of these issues [84]; consequently, only a computerized, robot-assisted MI procedure overcomes all of these drawbacks.

Interventional skills and ergonomics in computerized, robot-assisted MI procedures are improved through 3D visualization and increased freedom of movement, thereby fostering wider adoption of MI procedures [85,86,87]. The performance of such robotic procedures has evolved toward dynamic actions allowing for increased precision in finishing tasks and suturing, while employing MI techniques within autonomous interventions [88,89,90] or with human-in-the-loop intervention [91,92,93].

The preceding analysis confirms that patient comfort depends on the degree of tissue invasiveness, the precision of the procedure, the duration of the intervention, the healing time, etc., as well as on staff skill, ergonomics, and ease of execution. A safe, autonomous MI procedure [94]—utilizing sensing and visualization devices that complement or replace certain human skills while allowing for potential human intervention—can be performed using intelligent digital technologies. Thus, the targeted positioning accuracy and visualization capabilities can be achieved through image-guided robotic procedures, which enhance the surgeon’s skills and improve their posture throughout the intervention [4,9,95,96]. Furthermore, this type of procedure is particularly well suited for complex surgeries [97,98,99,100] or targeted drug delivery [101,102,103], both of which preserve surrounding healthy tissue.

The various robotic interventions mentioned previously—robot-assisted laparoscopy, computer-controlled robotics, and image-guided robotics—each require a tailored actuation methodology that takes into account characteristics such as positioning accuracy, movement resolution, resistance to EMI, and so on. Furthermore, the current category of actuated robotic joints used in medical interventions employs methods such as cyclic repetition and/or stepping operation, enabling longer travel ranges and a greater number of degrees of freedom [104]; these devices include stepper actuators [105,106] and ultrasonic actuators [107], most of which are suitable for medical interventions.

In the aforementioned field of safe, image-guided autonomous robotics, safety depends not only on the characteristics of the procedure but also on its duration, which is closely linked to the scanner’s operating mode. Indeed, during medical procedures, the duration of imaging necessitates the exclusive use of non-ionizing scanners—specifically magnetic resonance imaging (MRI) and ultrasound [97,98,99,100]—as interventional robotic instruments operate inside or in the immediate vicinity of the scanner. MRI technology is increasingly used for surgical interventions and drug delivery, primarily due to its ability to distinguish tumors (or, more generally, pathological areas) from healthy tissue during procedures such as tumor ablation [108,109,110,111] or drug administration [4,9]. Furthermore, it is applicable to all tissue types, unlike ultrasound, which is limited to tissues free of air and bone. It should be noted that the aforementioned characteristics of piezoelectric robotic actuation are fully compatible with both MRI and ultrasound technologies. Details regarding MRI compatibility will be addressed in the following section.

#### 4.1.3. Case of MRI-Guided Robotic Actuation

This section focuses on piezoelectric actuation systems designed for MRI-assisted robotic interventions. Beyond its innovative nature, this approach leverages the characteristic properties of piezoelectric materials, taking into account the specific environmental constraints associated with introducing foreign objects—such as the robotic system used here—into the MRI environment.

As indicated in the previous section, MRI offers the advantage of being able to distinguish tumors from healthy tissue and working with all types of tissue. However, its operation requires an environment free from EMI. This entails shielding against exposure to external electromagnetic fields (EMF) and excluding any EMF-sensitive materials from the scanner structure. In the absence of these essential precautions, serious disruptions would affect its operation and impair the image [4,9]. Consequently, robotic devices integrated into the scaffold—in the vicinity of the relevant body tissues—must generally be MRI-compatible; that is, they must be free of EMF-sensitive materials, such as magnetic materials or bulk conductive materials. Most robotic mechanisms, including robot structures and interventional instruments, can be built from MRI-compatible ingredients. In the realm of robotic actuation applications, few high-performance actuators offer such compatibility—unlike pneumatic and piezoelectric actuators. As previously noted, the latter outpace others in several respects, particularly regarding responsiveness [4,9,24,95,96] compared to pneumatic models, while also being potentially MRI-compatible. There are MRI-compatible piezoelectric actuation systems with varied characteristics in terms of shape, composition, construction, and use [112,113].

The literature presents various examples of stepping actuation [105,106], multi-DOF systems operating on the “stick-slip” principle [104] with flexible joints, biomimetic actuators replicating body movements [114,115,116], as well as ultrasonic standing-wave actuators [107] for linear and multidimensional displacement at the miniature scale [117,118,119]. Furthermore, examples of piezoelectric robotic hands designed for manipulating objects ranging from the micrometer to the millimeter scale, as well as adaptive miniature piezoelectric robots, are presented in references [120,121,122,123]; cases involving micro-positioning robotic surgical instruments and image-guided biopsies with optical coherence tomography (OCT) control are described in references [124,125]. Finally, examples concerning the role of robotics in image-guided interventions and the field of medical robotics are provided in references [126,127].

**Figure 3:**
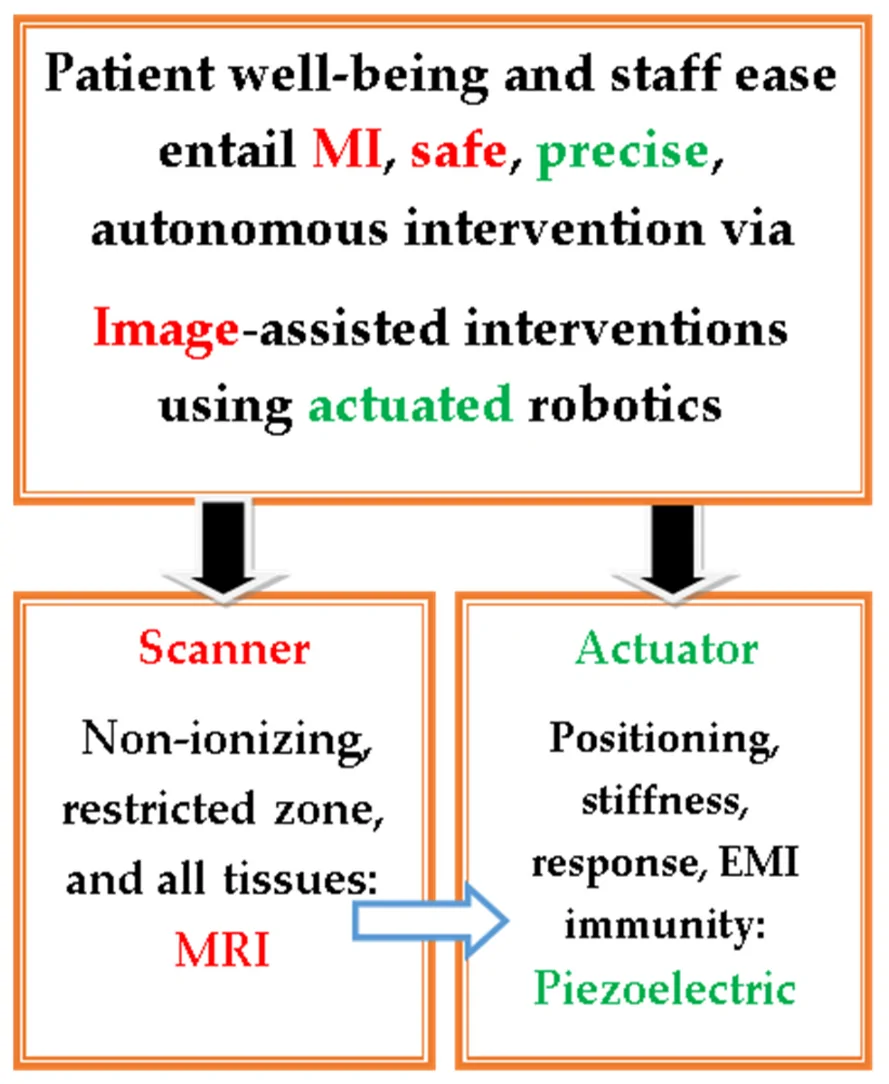
Summarized selection strategies relative to scanner and robotic actuation in an image-guided robotic intervention [13].

Figure 3 summarizes the association between safety and precision linked to the scanner and robotic actuation in the context of image-guided robotic intervention [13].

### 4.2. Sensing, Assisting, and Health-Monitoring Wearable Devices

Wearable devices can perform tasks such as detection [128,129], diagnosis [130], monitoring [131,132,133], control [134], and assistance [135]. Piezoelectric pressure sensors are used in many such applications. They are characterized by high sensitivity and excellent linearity, making them appropriate for various sensing needs, particularly for capturing dynamic signals. Consequently, they are employed for precise wrist pulse acquisition [34], the assessment of tremors associated with Parkinson’s disease [35], and MI robotic interventions [36,37]. As for assistance functions, these are carried out through active tasks—as previously mentioned—such as stimulating organ function [44,45,46,47,48] or assisting limbs with sensorimotor impairments [6,7,8]. These active tasks can be achieved using integrated robotic systems directly actuated by piezoelectric patches [20,21,57,58,59,60,61].

#### 4.2.1. Multifunctional Flexible Piezoelectric Wearable Devices

Piezoelectric materials are particularly well suited for portable devices, thanks to their ability to perform multiple functions and enable the design of flexible equipment. Indeed, they exhibit direct and inverse conversion phenomena, corresponding to sensing and actuation, respectively. Although this bidirectional electromechanical conversion is not unique to piezoelectricity, these materials offer exceptional electromechanical efficiency and remarkable scalability compared to competing solutions, thereby facilitating miniaturization. Consequently, they are widely used for sensing, actuation, or applications integrating both functions [136]. Regarding the design of flexible devices, wearable units can—in addition to their sensing and actuation functions—harness energy harvesting from human body movements using flexible piezoelectric materials (FPMs) that are particularly well suited to large deformations [137].

**Figure 4:**
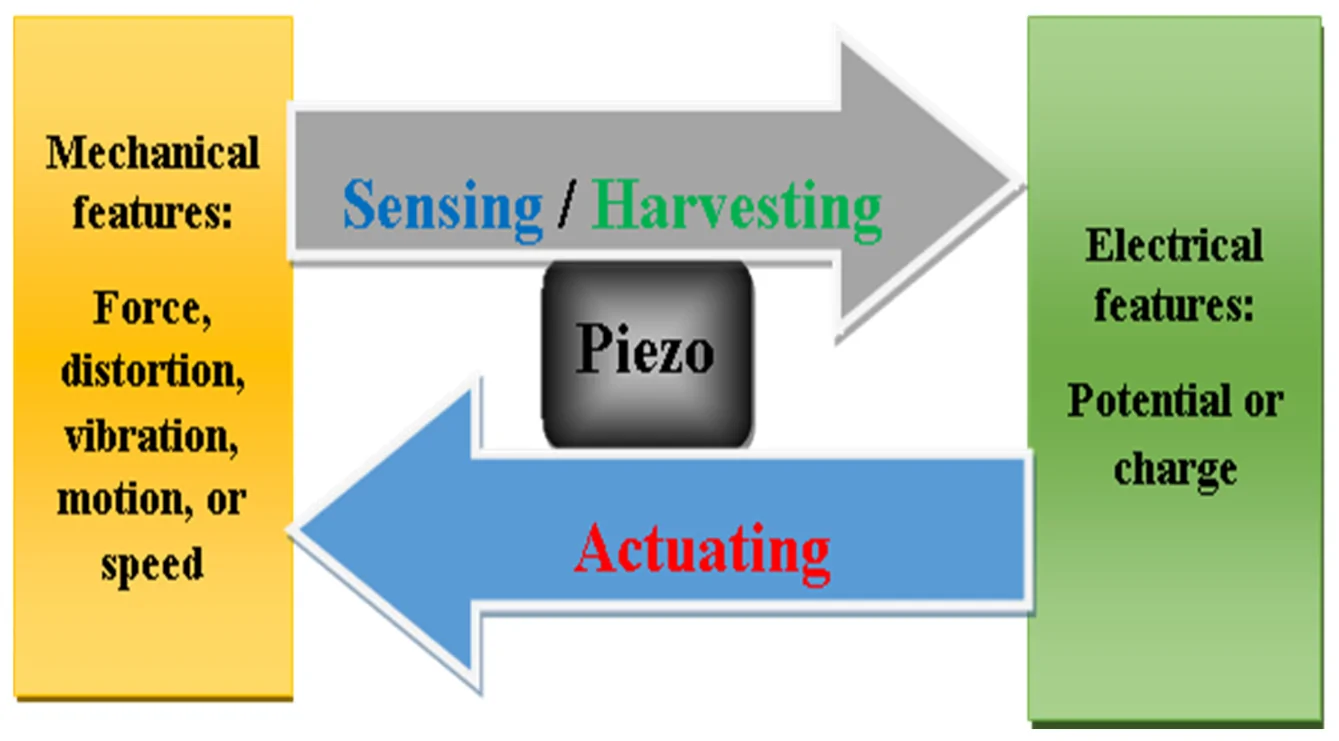
Schematic illustration of a multifunctional piezoelectric device performing sensing and harvesting functions as well as the inverse actuating function [24].

A multifunctional device is generally capable of integrating various tasks using its own components. Such a combination can result in a more compact device. Multifunctional integration naturally combines sensing and energy harvesting functions, thereby creating a self-powered sensor. Figure 4 schematically illustrates a multifunctional piezoelectric device that performs sensing and energy harvesting functions via mechanic–electric conversion, as well as the inverse function for actuation [24].

In the case of FPMs, mechanical flexibility is a key feature of wearable devices, which are inherently miniaturized and lightweight. This flexibility extends beyond the FPM itself to include its substrates and electrodes, thereby enabling the creation of a flexible, multifunctional, and reliable piezoelectric device. This body-conformable flexibility aligns closely with dynamic human movements [137].

Regarding wearable energy harvesting, it can be associated to heart rate [138,139], respiration [140,141], pulse [142], and gastric cavity deformation [143].

FPMs can be produced using various technologies, primarily polymers [144,145], composites [146,147], and inorganic thin films [148,149]. Although all three types of FPMs possess the required piezoelectric and flexible properties, their performance characteristics differ significantly. Polymers are inherently organic and fully flexible but exhibit the weakest piezoelectric properties; conversely, inorganic films offer superior crystalline piezoelectric characteristics but are mechanically more fragile; finally, composites—resulting from a crystal-polymer combination—exhibit intermediate behavior, where flexibility and piezoelectric properties can be tuned through mechanical design or mixture proportions.

These distinctive behavioral characteristics guide the selection of appropriate applications for each type of FPM [150]. For example, polymers lend themselves to motion control on complex surfaces or substrates, whereas inorganic thin films better meet the need for enhanced sensitivity. Similarly, FPMs in the form of composites or thin films are better suited for actuation and energy harvesting applications. Numerous studies focusing on applications involving piezoelectric wearable devices multifunctionality, flexibility, or both, have been published recently. The following section presents examples of this work.

#### 4.2.2. Applications of Multifunctional/Flexible Piezoelectric Wearable Devices

Numerous recent studies have been published on wearable piezoelectric devices, highlighting their multifunctional and flexible nature as well as various aspects such as structural operation, activity monitoring, environmental behavior, and interactive applications, among others.

Regarding structural operation, the literature reports on various applications, including energy harvesting [151,152,153], sensing coupled with energy harvesting [154,155,156], sensing and actuation [157,158], and general sensing [159,160,161,162]. It is worth noting that, in addition to piezoelectric devices, other types of flexible wearable devices can be produced, such as flexible pressure sensors based on shape-memory polymers [163].

Concerning activity monitoring, various applications focus on continuous monitoring with various activities such as implantable devices, human motion monitoring, finger rehabilitation and more [164,165,166,167,168,169,170,171,172,173], physical and sports activity tracking [174,175], and electronic skin (“e-skin”)—a flexible, stretchable, and self-healing electronic system [176,177].

With respect to environmental behavior, various studies have been published on biodegradability [178,179,180], non-invasive sensing [181,182], and lead-free applications [183,184].

As for interactive applications, different studies focus on robotics [185,186], human-machine interaction [187], and combined (all-in-one) operations [188].

Furthermore, other applications encompass topics such as the direct use of piezoelectric materials [189], electromechanical transduction [190], and, finally, work related to material studies [191,192].

## 5. Integration into Medical Systems Driven by Intelligent Digital Tools

Robotic actuation in medical interventions, as well as wearable devices for sensing, assistance, and health monitoring—utilizing the piezoelectric actuators and sensors mentioned in the previous section—can be integrated into autonomous medical systems driven by intelligent digital tools. This approach would combine the precision of piezoelectric devices with a reduction in the constraints inherent to medical procedures, while facilitating decision-making or enhancing human-machine interaction, all managed by intelligent digital tools.

Many medical interventions or procedures, much like industrial processes, are designed to operate autonomously. An autonomous medical intervention typically encompasses the medical instrument(s), the target organ or body part (within its environment), and the intervention’s protocol and constraints; the entire system operates within a closed-loop control framework, utilizing sensors for detection and actuators for execution. Such interventions can be complex due to the interdependent behavior of the components and their interactions. Furthermore, detection and control processes may involve uncertainties. Finally, the intervention may be exposed to unforeseen external threats.

To ensure the reliability of the intervention, it is necessary to monitor these disruptive factors: complexity, uncertainties, and external threats. Moreover, depending on the nature of the intervention, decision-making capabilities may be required. Finally, even with autonomous procedures, human interaction remains essential when the situation demands it.

Thanks to current advances in intelligent digital tools, the aforementioned functions—monitoring, decision-making, and human-machine interaction support—can be performed by DT [26,27], AI [28,29,30], and xR technologies [31,32,33], respectively. It should be noted that the term xR encompasses augmented reality (AR), virtual reality (VR), and mixed reality (MR), while AI includes specialized fields such as machine learning (ML) and deep learning (DL).

These intelligent digital tools can be used, for example, in the scenario discussed in Section 4.1.3 regarding MRI-guided robotic intervention; this scenario leverages the characteristic properties of piezoelectric materials while accounting for the specific environmental constraints associated with introducing foreign objects—such as the robotic system used here—into the MRI environment. Such use of intelligent digital tools enables supervision of this controlled autonomous procedure—potentially enhanced by AI-based assistance—based on the DT concept, as well as support via xR technologies for interventions involving human interaction (a “human-in-the-loop” approach).

### 5.1. DT Monitoring of an MRI-Guided Controlled Robotic Intervention

The control precision of such an autonomous robotic intervention would depend on the aforementioned disturbing factors as well as on constraints inherent to the intervention, such as MRI-compatibility and patient-specific characteristics. Reliable monitoring of this autonomous procedure enables the management of these parameters. This can be achieved by aligning the physical-virtual pair that constitutes a DT [193].

In essence, a DT consists of a physical element, a virtual replica of that element, and a near-real-time, bidirectional data exchange between the two. Thus, the DT is conceived as a combination of the physical entity and its digital representation, establishing a bidirectional interaction. This setup is implemented for the supervision of complex processes [194] in the form of a physical–virtual pair that enables self-adaptation actions. The physical element transmits sensed and processed information to the virtual element, while the latter communicates control instructions to the former. These adjustment mechanisms, combined with complexity management, help reduce uncertainties within the pairing as well as unforeseen threats that disrupt robot control dynamics. Specifically, when matching data from the physical element to the virtual element, the data are adjusted using external means—such as the Internet of Things (IoT)—and information derived from the process’s operational history. The result, obtained following a data learning and analysis phase, is transmitted to the virtual element along with an appropriate reduced-order model of the process. Indeed, rapid matching of the DT requires a model with low computational time while maintaining a realistic representation of the process. It should be noted that a more complex process entails a more complex full coupled model and, consequently, longer computation times.

The concept of DT has been progressively embraced in the healthcare sector, covering patient treatment, solution development, and the long-term management of health conditions. Comprehensive reviews have described its applications and implementation in treatment, monitoring, and care management [195,196,197,198,199,200].

DT monitoring is generally implemented within the context of personalized therapies, whether involving medical treatments or interventions. It can be used for medical staff training, for predictions based on physical phantoms and their virtual counterparts, or for setups linking a real patient to a virtual model—involving autonomous registration complemented by human supervision. This collaboration between staff and robotics enables an innovative approach to MRI-guided interventional monitoring, thereby reducing patient risk while ensuring the reliability of the procedures performed by the staff [201,202,203].

### 5.2. Smart Digital Tools and Personalized Tasks

The use of intelligent digital tools—such as DT, AI (including ML and DL), and xR (encompassing AR, VR, and MR)—combined with medical therapeutic supervision, enables the execution of various other tasks, such as procedure planning, staff training, therapeutic investigation, tutoring, and more.

Medical interventions necessitate advance planning to verify and adjust intended tasks, which can be attained through physical phantoms and their digital replicas in DT tool. These preparations incorporate the intervention itself as well as patient-specific data [204], while ensuring the appropriate adaptation of procedure-related parameters (such as scanner settings and compatibility [205], the control and management of robotic systems, etc.). Furthermore, such personalized planning permits staff to evaluate the choices made and become aware of potential errors in judgment [206,207].

The literature describes various medical applications utilizing intelligent digital tools, particularly in the fields of cognitive control [208], interventional training [209], medical education and learning [210,211], surgical advancements [212], rehabilitation [213,214], ethical frameworks regarding the use of digital tools [215], and various AI-assisted clinical or care applications [216,217,218,219].

**Figure 5:**
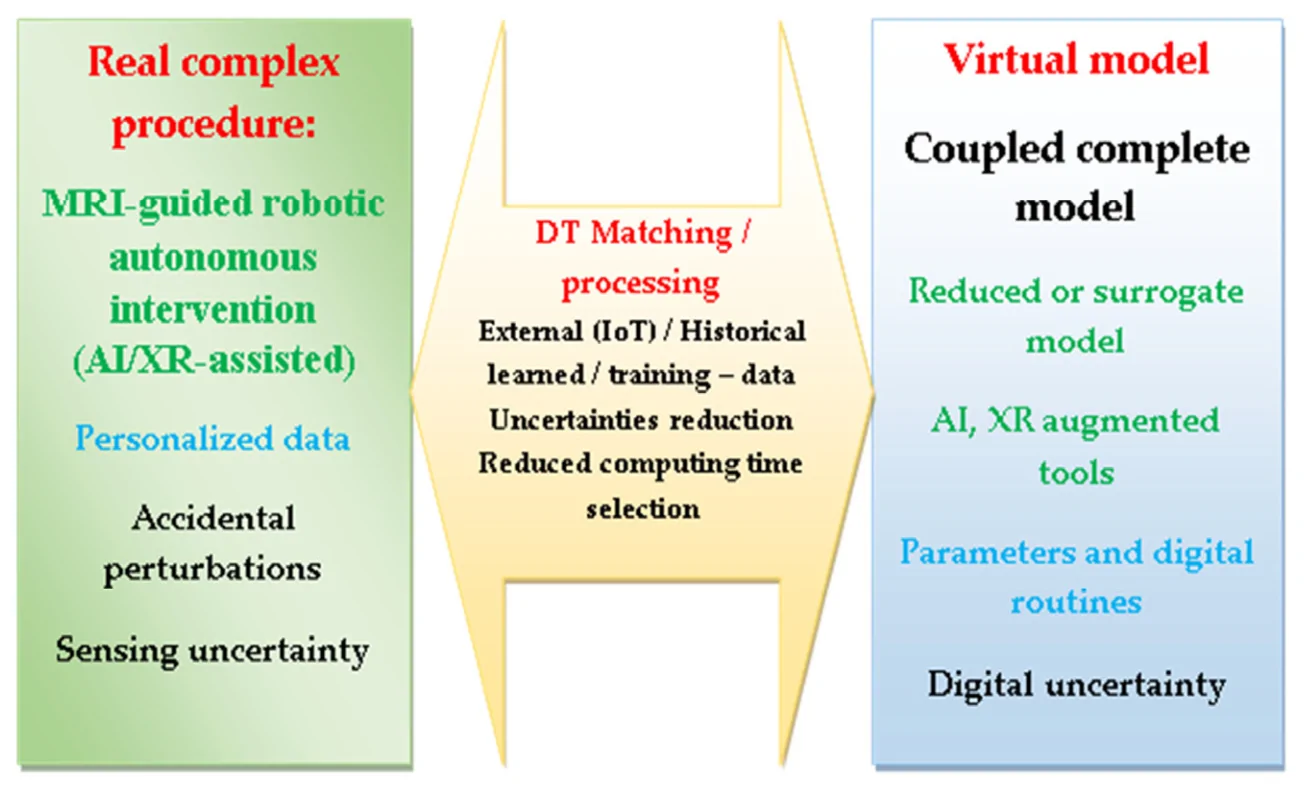
Summarized schematics of DT monitoring of an MRI-guided robotic intervention with AI and xR digital tools [12].

**Figure 6:**
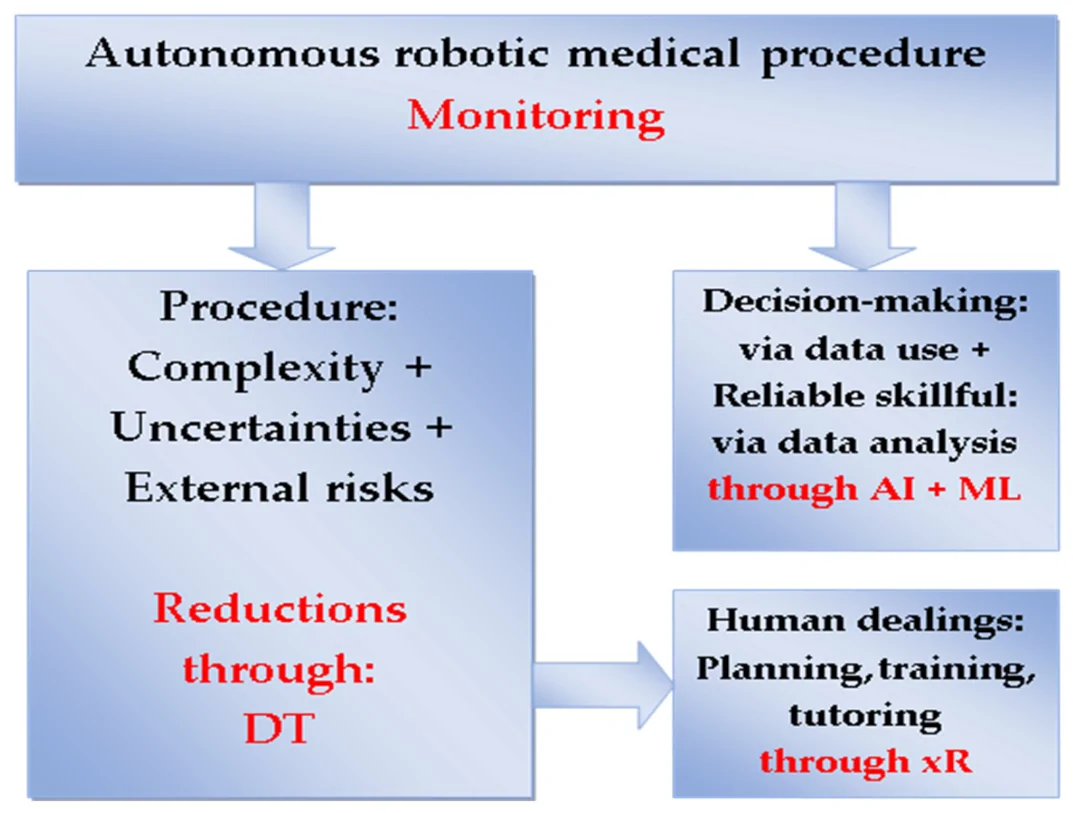
Summarized complementary roles of DT, AI, and xR in the management of autonomous robotic medical procedures.

Figure 5 schematically illustrates the characteristics of DT-based monitoring of an MRI-guided robotic intervention, leveraging digitally augmented AI and xR tools [12]. The complementary roles of DT, AI, and xR are summarized in Figure 6.

## 6. Discussion

Building on the investigations and analyses presented in the preceding sections, several practical issues warrant further exploration; these concern medical monitoring, assistance, and interventions, as well as general concepts such as EMI, smart digital tools, etc., without, however, being limited to the integration of smart piezoelectric devices. Proposals for future research avenues are also presented.

### 6.1. EMI Immunity of Medical Devices

EMI immunity has been mentioned several times in the preceding sections, particularly regarding MRI-compatibility. A device’s vulnerability to EMI is linked to its exposure to external EMFs; these induce fields within the device’s sensitive components—such as electrical conductors—or disrupt the device’s intrinsic field. In either case, the device’s operation would be impaired. Such immunity can generally be guaranteed through fitting design or the use of shielding. Design focused on immunity to EMI relies on reducing parasitic fields emitted by the source, manufacturing the target medical device from materials insensitive to EMFs, and/or protecting the device with appropriate shielding.

The most common shielding technique involves placing a sheet of conductive material between the source and the device—and often around it—offering a simple and cost-effective solution. Currents induced in the conductive sheet by incident EMFs create a shielding effect via the skin effect, thereby preventing the field from penetrating the device. However, these induced currents cause heating due to energy dissipation; the resulting temperature rise depends on the nature and duration of the exposure, as well as the physical characteristics of the sheet.

For wearable devices, heating of the shield can impair device operation and lead to conductive heat transfer to body tissues. This issue can be mitigated by modifying the electrical conductivity characteristics of the shield through the use of smart shielding materials.

Such modification can be attained by using multifunctional components designed for low-reflectivity shielding, thereby reducing the significant EM reflection caused by the material’s high conductivity. Furthermore, a specific manufacturing process capable of reducing the material’s power reflection coefficient can also be employed [220,221,222,223,224]. Shielding effectiveness can be verified through EM compatibility (EMC) analysis [16,17].

### 6.2. Physical Complexity Monitoring and Model Reduction in DT

As indicated in Section 5, medical interventions can be complex due to the interdependent behavior of the components and their interactions. Indeed, these components involve various physical and biological phenomena that may interact and exhibit interdependent behaviors during the intervention. Mathematically modeling this interdependence and these interactions suggests the use of a comprehensive coupled model that integrates the equations governing the various phenomena. In the general case, such coupling may involve behavioral nonlinearities, distinct (though potentially similar in order of magnitude) phenomenological time constants, and complex geometries; these factors necessitate the simultaneous solution of the equations at the local scale. The resulting solution procedure relies on a discretized local strong coupling, which entails significant computational time. Furthermore, it should be noted that the more complex the intervention is, the more complex the solution of the coupled model becomes.

Managing this complexity through DT monitoring involves matching the physical procedure to its virtual replica, which incorporates the full coupled model. As mentioned in Section 5.1, an appropriate reduced-order model or surrogate model [225,226,227] of the process is required for the virtual component of the DT. Indeed, rapid DT matching is incompatible with the use of the full coupled model, even if the latter faithfully represents the actual physical procedure. In general, it is necessary to execute a model sensitivity analysis pursued by a model reduction technique to reduce computational time while preserving the physical reality of the problem and retaining only those characteristics essential for an accurate physical representation.

### 6.3. Validation of Studies and Pathway to Clinical Personalization

The transition from research to clinical implementation depends on both digital skills linked to technological advances and structured policies regarding digital health solutions.

Regarding the link between digital skills and technological advances, this relationship has fostered effective progress based on the adoption of these technologies [228,229]. In the areas of monitoring, assistance, and intervention, this link has proven crucial, particularly in the context of the recent pandemic. Indeed, it helps facilitate the digitalization of healthcare [230,231,232,233], thereby improving health outcomes and workforce capabilities.

As for the policy framework governing digital health solutions, the focus of staff, patients, and designers is increasingly shifting toward the patient, holistic design, transparency, and sustainability [234,235,236]. Furthermore, multifunctional AI-based smart devices, smart materials, and energy management systems enable real-time detection [237]. Additionally, biocompatibility, durability, and treatment consistency facilitate reliable diagnostics, thereby enhancing clinical practice [238,239].

Thus, numerous recent contributions mark the transition from research to clinical implementation, such as AI-assisted clinical procedures [240,241,242], stimulation and brain-machine interfaces [243,244,245], wearables enhanced by digital skills [246,247,248,249,250,251], and integrated tools [252,253,254]. Specific clinical applications are also evident, including diagnostics [255], remote monitoring [256], environmental treatment [257], spinal cord injury management [258], and issues regarding equipment costs [259]. Finally, various advances have been initiated, such as shape memory alloy actuators for upper-limb prosthetic application [260], AI in cardiothoracic interventions [261], self-oriented gradient ionic skins for dual-function EM shielding and self-powered sensing [262], high-entropy alloys sensing materials for next-generation flexible electronics [263], and piezoelectric actuators for compliant mechanisms [264].

### 6.4. Identifying of Piezoelectric Devices and Materials by Application

The analysis presented in the preceding sections of the article, focusing on robotic and wearable medical systems, examined various types of piezoelectric devices made from diverse materials. The classification of piezoelectric sensing and actuation mechanisms falls outside the primary scope of this review. Nevertheless, to better guide readers, several key references addressing these classifications—such as [52,105,107]—may be consulted for detailed taxonomies.

The device type and the material used are closely linked to the intended application—specifically, robotic and wearable systems—encompassing both robotic actuation and wearable sensors. Piezoelectric robotic actuation can be achieved through self-propelled miniature structures incorporating the piezoelectric material or via joint actuators that convert piezoelectric deformation into indirect motion. Piezoelectric pressure sensors are commonly used in wearable devices; piezoelectric materials are particularly well-suited for this purpose due to their ability to perform multiple functions and enable the design of flexible equipment.

Self-propelled miniature medical robotic structures [57,58,59,60,61] consist of elements such as beams, plates, rods, or conduits incorporating piezoelectric actuation devices (bonded, embedded, integrated, etc.). Furthermore, cyclic and/or stepping operation enables greater displacement amplitudes and an increased number of degrees of freedom through the use of stepping and ultrasonic actuators [105,106,107] at the robotic joints used during medical interventions.

In the case of FPMs, mechanical flexibility is a key feature for wearable devices, enabling the creation of a flexible, multifunctional, and reliable piezoelectric device. This flexibility, which allows the device to conform to the body’s contours, aligns perfectly with dynamic human movements [137]. FPMs can be fabricated using various technologies, primarily involving polymers [144,145], composites [146,147], and inorganic thin films [148,149].

Regarding the materials employed, these consist of piezoelectric ceramics or composite piezoelectric materials. Piezoelectric ceramics, which serve as essential drive components for actuators, require a high electric field to generate significant field-induced strain due to their high coercive field. However, high-coercive-field piezoelectric ceramics are difficult to integrate into practical products. Consequently, it is crucial to develop a type of piezoelectric ceramic capable of exhibiting substantial field-induced strain and low hysteresis under a low-intensity electric field [265]. In the case of composite piezoelectric materials, it has been demonstrated that the electric-field-induced strain in 0-3 type composite ceramics is increased by 161% compared to that of non-composite ceramics, while hysteresis is reduced by 230% relative to the latter [266].

Here are a few practical examples illustrating how these technologies are integrated into real-world medical systems. The first example is related to a wearable insulin delivery system for artificial pancreas. It consists of a piezoelectric micro pump, which adopts a dual-chamber series structure with asynchronous drive and is made of a biocompatible polymer. The proposed insulin delivery system has a simple form, low failure rate, and adaptability to common voltage working conditions. Animal experiments show that the proposed wearable insulin delivery system has a significant hypoglycemic effect on large-eared white rabbits, verifying its feasibility in practical applications [60]. The second application concerns the design and fabrication of a piezoelectric micro pump that operates based on the action of a traveling mechanical wave deforming the bottom wall of a flexible fluid-filled channel. A simple structure consisting of a channel and reservoirs (see Figure 2) was designed to test the device. Several flow rate and back-pressure measurements were performed to evaluate the prototype’s performance at various wave amplitudes. The medical application of such a device enables the precise and controlled delivery of fluids [61]. The third application focuses on the feasibility of designing and implementing robust position control for piezoelectric motors of various topologies. The control technique must meet the specific precision and robustness requirements of robotic applications while satisfying the simplicity criteria necessary for practical implementation. This work highlights the advantages and limitations of each piezoelectric positioning system in the context of robotic applications, particularly for MRI-compatible actuation during robot-assisted medical interventions [19].

### 6.5. Examples of the Integration of Smart Digital Tools into Piezoelectric Medical Devices

This section is dedicated to practical examples illustrating how smart digital technologies are integrated in medical applications involving piezoelectric devices. This is the case, for example, with flexible piezoelectric devices designed for wearable medical equipment; while flexible piezoelectric nanogenerators enable wearable technologies to provide continuous health monitoring, their reliance on batteries limits their long-term use. These technologies address such challenges by enabling autonomous operation. When integrated into such piezoelectric sensors, AI enhances signal processing accuracy, anomaly detection, and adaptive energy management. Ultimately, this enables the automatic recognition of physiological patterns and state changes in various biomedical applications, such as cardiovascular and neuromuscular monitoring [267,268,269]. DT-assisted surgery serves as another pertinent example, as computer- (or image-) assisted robotic surgery has become essential to modern precision medicine for the specific planning, management, and execution of procedures. Indeed, robotic precision can be achieved through piezoelectric actuation. Digital twin technology proves particularly valuable due to its ability to create a patient-specific replica of the procedure while enabling medical staff to monitor its progress in real time. Combining these two tools enhances the precision of minimally invasive surgery, thereby facilitating a successful and pivotal clinical translation [270,271].

### 6.6. Perspectives on Piezoelectric Tools and Medical Robotic and Wearable Systems

Future prospects regarding the topics addressed in this contribution fall into two categories: piezoelectric tools and medical systems. The first aspect concerns the environmental conditions under which these tools are used, while the second focuses on how the systems account for body tissues.

#### 6.6.1. Biocompatibility, Biodegradability, and Non-Toxicity in Integrated Devices

This section focuses on the environmental biosafety associated with the use of piezoelectric devices. Various medical applications involve monitoring, assistive, or interventional procedures performed within or near living tissues. Most of these interventions require non-toxicity and biocompatibility, or even—in certain circumstances—biodegradability. Furthermore, the presence of biological piezoelectric properties in parts of the body (such as skin, tendons, bones, and other tissues) highlights the potential of biomaterials in biomedicine to enhance or replace biological functions [272,273]. Additionally, the growing use of lead-free piezoelectric materials in various applications [183,184] further demonstrates this lack of toxicity. Thus, biocompatibility and non-toxicity—and, consequently, the absence of immune rejection—ensure overall biosafety. The drive to improve therapies and address patients’ long-term needs [274,275] is fostering research into piezoelectric biomaterials and lead-free materials for medical treatments.

#### 6.6.2. Complexity and Tissue Dynamic Behavior in Real Time Involvements

Several aspects of this contribution present physical and mathematical representations of biological tissues, particularly within the context of DT-based monitoring systems. These representations take the form of physical and virtual phantoms (models)—often based on approximate static behavioral characteristics—that enable the accurate consideration of numerous realistic scenarios. In reality, body tissues consist of soft matter permeated by the fluids essential to their function. This composition gives rise to dynamic mechanical displacements and deformations that cannot be faithfully represented by the aforementioned static behavior. This is the case, for instance, with skin [276,277], where a static tissue representation proves inadequate, necessitating dynamic mechanical analysis (DMA). The same applies to the robotic manipulation of deformable objects [278] or the autonomous, DT-assisted manipulation of soft tissues [279]. DMA plays a pivotal role in biomechanics by enabling the representation of tissue viscoelastic properties, thereby facilitating the development of real-time, tissue-specific, multi-scale models. Research into these mechanical representations is of major importance and forms part of the multiphysics coupling of tissues with other phenomena involved in medical treatments.

#### 6.6.3. Perspectives Integrated in the Contribution Background

**Figure 7:**
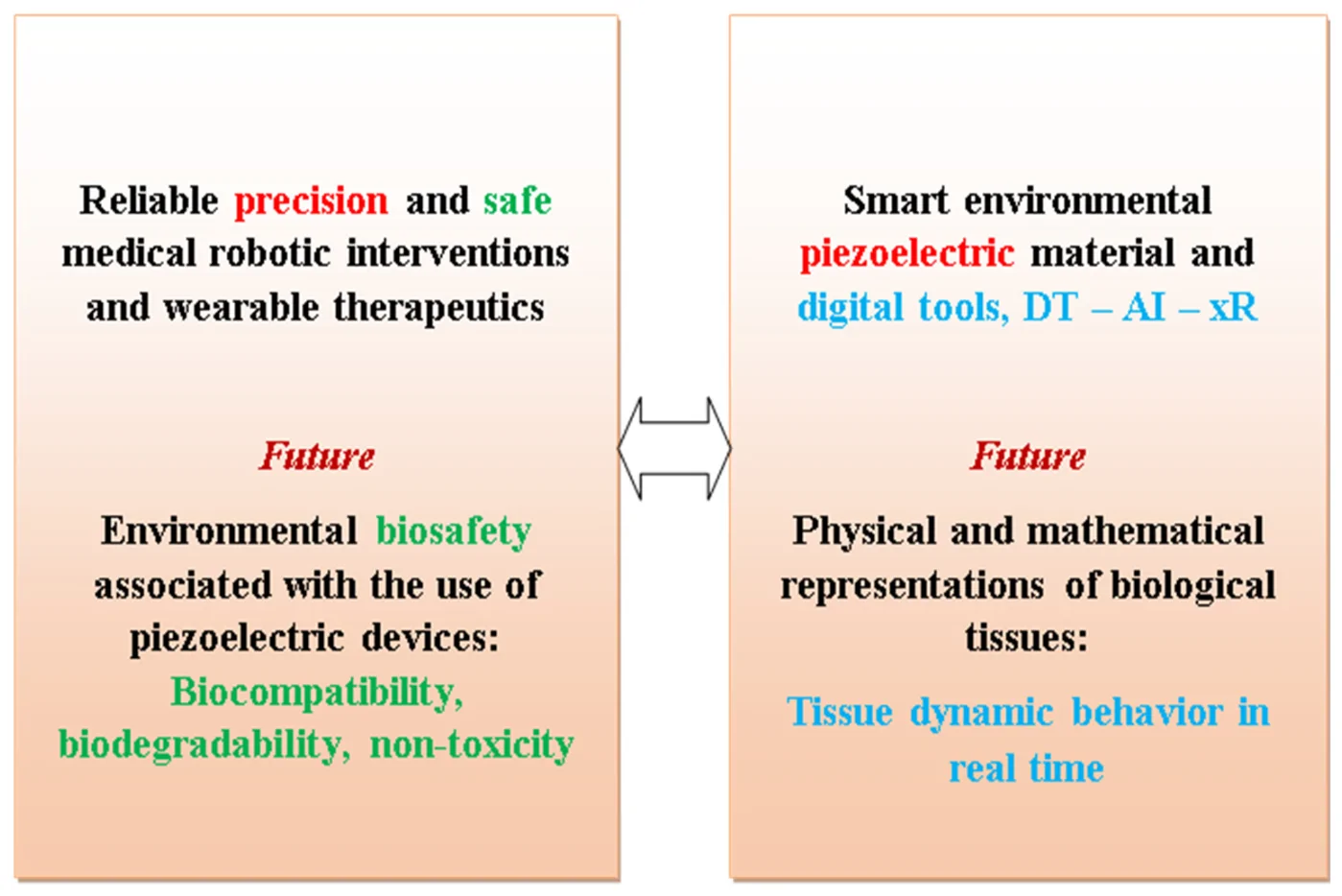
Future projections in the context of the objectives of this contribution and the means of achieving them.

Figure 7 places future projections in the context of the objectives of this contribution and the means of achieving them.

## 7. Conclusions

The analyses and studies presented in this article highlight the potential of piezoelectric biomedical devices for actuation and sensing functions within systems driven by smart digital tools. These devices enable not only reliable, safe, and highly precise medical interventions but also observation, monitoring, and support within personalized therapeutic approaches utilizing wearable devices—all controlled by smart digital tools. Ultimately, the key conclusion is that combining smart materials with smart digital tools represents a particularly relevant approach, especially given that the material in question significantly outperforms its competitors in the context of the study at hand. This combination can allow for the integration of piezoelectric device precision with a reduction in the constraints inherent to medical procedures, while facilitating decision-making or enhancing human-machine interaction—all managed by smart digital tools.

Two avenues for future research on the article’s topic suggest further investigation into the following points (see details in Section 6.6):

- Enhancement of environmental biosafety associated with the use of piezoelectric devices. Thus, biocompatibility and the absence of toxicity—and, consequently, the absence of immune rejection—ensure overall biosafety.
- Improvement of physical and mathematical representations of biological tissues. These enable the modeling of tissue viscoelastic properties, thereby facilitating the development of real-time models specific to each tissue type.

## Figures and Tables

**Table 1:** Comparison of performances of different actuating devices involved in medical interventions [24].

Actuator	Resolution	Response Time	Driving Force	MRI Compatibility
piezoelectric	Nanometer	Microsecond	Newtons	High
electromagnetic	Tens micrometers	Millisecond	Newtons	Low
hydraulic	Micrometer	Millisecond	Newtons	High
shape memory alloy	Nanometer	Second	Newtons	Medium

## Data Availability

Data will be made available on request.
